# The First Report of Polymorphisms and Genetic Characteristics of the Shadow of Prion Protein (*SPRN*) in Prion Disease-Resistant Animal, Chickens

**DOI:** 10.3389/fvets.2022.904305

**Published:** 2022-06-17

**Authors:** Yong-Chan Kim, Hyeon-Ho Kim, Byung-Hoon Jeong

**Affiliations:** ^1^Korea Zoonosis Research Institute, Jeonbuk National University, Iksan, South Korea; ^2^Department of Bioactive Material Sciences, Institute for Molecular Biology and Genetics, Jeonbuk National University, Jeonju, South Korea

**Keywords:** chickens, prion, PRNP, SPRN, SNP, polymorphism, BSE

## Abstract

Prion diseases are irreversible neurodegenerative disorders caused by the aggregated form of prion protein (PrP^Sc^) derived from the normal form of prion protein (PrP^C^). Previous studies have reported that shadow of prion protein (Sho) interacts with prion protein (PrP) and accelerates the conversion of PrP^C^ to PrP^Sc^. In addition, genetic polymorphisms of the shadow of the prion protein gene (*SPRN*) are related to the vulnerability of prion diseases in various hosts. However, to date, polymorphisms and genetic features of the *SPRN* gene have not been investigated in chickens, which are prion disease-resistant animals. We investigated genetic polymorphisms of the *SPRN* gene in 2 breeds of chickens, i.e., Dekalb White and Ross, using amplicon sequencing. We analyzed genotype, allele and haplotype frequencies and linkage disequilibrium (LD) among the genetic polymorphisms. In addition, we compared the amino acid sequences of Sho among several prion-related species to identify the unique genetic features of chicken Sho using ClustalW. Furthermore, we evaluated the N-terminal signal peptide and glycosylphosphatidylinositol (GPI)-anchor using SignalP and PredGPI, respectively. Finally, we compared the number of *SPRN* polymorphisms between prion disease-resistant and prion disease-susceptible animals. We identified 7 novel single nucleotide polymorphisms (SNPs), including 1 synonymous SNP in the open reading frame (ORF) of the chicken *SPRN* gene. We also found significantly different genotypes, allele frequencies and haplotypes between the 2 chicken breeds. In addition, we found that the interaction regions between Sho and PrP and the NXT glycosylation motif were conserved among all species. Notably, sequence similarity was extremely low in the N-terminal and C-terminal regions between mammals and chickens. Furthermore, we found that chicken Sho was the longest N-terminal signal peptide, and the amino acids of the cutting site of chicken are different from those of mammals. Last, unlike other species investigated, omega-site and signal sequences of the GPI-anchor were not found in chickens. To the best of our knowledge, this is the first report of genetic polymorphisms of the *SPRN* gene in chickens.

## Introduction

Prion diseases are fatal and infectious neurodegenerative disorders caused by the misfolded toxic form of prion protein (PrP^Sc^) originating from a benign form of prion protein (PrP^C^). Although the conversion process of PrP^C^ to PrP^Sc^ is still unclear, several factors that play a pivotal role in the conversion have been identified thus far (1–5). Among these factors, previous studies have reported that the shadow of prion protein (Sho) interacts with prion protein (PrP) and accelerates the conversion of PrP^C^ to PrP^Sc^ (6). Sho is a member of the prion protein family, which contains PrP, Sho, prion-like protein (Doppel) and prion-related protein (Prt) and has a similar protein structure to PrP, including a repeat domain and glycosylphosphatidylinositol (GPI)-anchor (7). Since Sho is involved in the pathological process of PrP^Sc^, genetic polymorphisms of the shadow of the prion protein gene (*SPRN*) are related to susceptibility to prion diseases in various hosts (8–11). In humans, an insertion/deletion polymorphism at codon 46 of the *SPRN* gene was significantly associated with susceptibility to variant Creutzfeldt–Jakob disease (vCJD) (10). In addition, the 602_606insCTCCC polymorphism in the 3' untranslated region (UTR) of the caprine *SPRN* gene also showed a significant association with vulnerability to scrapie in goats (11). Furthermore, an insertion/deletion polymorphism of the bovine *SPRN* gene was identified in atypical bovine spongiform encephalopathy (BSE)-affected cattle (12). In contrast, horses, a prion-resistant animal, showed different 3-dimensional (3D) structures of Sho with additional alpha-helixes compared to prion-susceptible animals. In 3 breeds of horses, only synonymous single nucleotide polymorphisms (SNPs), which do not affect the 3D structure of the Sho, were found in the equine *SPRN* gene (13, 14). These studies indicated that genetic characteristics of the *SPRN* gene are significantly different between prion-susceptible and prion-resistant animals. Previous studies have reported that chickens showed perfect resistance to experimental BSE infection despite high sequence similarity with PrP of prion-susceptible animals (15). However, a clear mechanism for resistance to prion disease in chickens is unclear, and Sho, a major factor in PrP^Sc^ conversion, has not been investigated thus far.

In the present study, we investigated genetic polymorphisms of the *SPRN* gene in 2 breeds of chickens, i.e., Dekalb White and Ross chickens. We also examined genotype, allele and haplotype frequencies and analyzed linkage disequilibrium (LD) among the genetic polymorphisms. In addition, we carried out multiple sequence alignments among several prion-related species to identify the inherent genetic features of chicken Sho using ClustalW (16). Furthermore, we investigated N-terminal signal peptide and GPI-anchor using SignalP and PredGPI, respectively (17, 18). Finally, we compared the number of *SPRN* polymorphisms between prion disease-resistant species (horses, chickens) and prion disease-susceptible species (humans, cattle, goats and sheep).

## Materials and Methods

### Ethical Statements

Dekalb White and Ross breeds were obtained from a slaughter house in the Republic of Korea. All experiments were carried out following the Korea Experimental Animal Protection Act. All experimental procedures were accredited by the Institutional Animal Care and Use Committee (IACUC) of Jeonbuk National University (IACUC number: JBNU 2017–0030). All efforts were made to minimize suffering.

### Genomic DNA

Genomic DNA was obtained from 20 mg of tissue samples using the Labopass Tissue Genomic DNA Isolation Mini Kit (Cosmogenetech, Seoul, Korea) following the manufacturer's protocols.

### Genetic Analysis of the Chicken *SPRN* Gene

The chicken *SPRN* gene (Gene ID: BN000836.1) was amplified from the genomic DNA by polymerase chain reaction (PCR) using gene-specific primers (F: 5′-TGCTCACATTCAGTGGGTGC-3′, R: 5′-TCTGCATTCTCCCTGTTGGG-3′). The PCR mixture was composed of 2.5 μl of 10× H-star *Taq* reaction buffer, 5 μl of 5× band helper, 1 μl of each 10 mM dNTP mix, 1 μl of each primer (10 μM), 0.2 μl of H-star *Taq* DNA polymerase (BIOFACT, Daejeon, Korea) and DEPC water up to a total volume of 25 μl. The PCR was performed with the following experimental conditions: 98°C for 15 min for denaturation; 40 cycles of 98°C for 20 sec, 58°C for 40 s and 72°C for 1 min for annealing and extension; and 1 cycle of 72°C for 5 min for the final extension. PCR was performed using a C1000 Touch Thermal Cycler (Bio–Rad, Hercules, California, USA). The PCR products were analyzed by an ABI 3730 sequencer (ABI, Forster City, CA, USA). The sequencing peaks were visualized by Finch TV software (Geospiza Inc., Seattle, WA, USA). We performed genotyping of each nucleotide with Q>40.

### Statistical Analyses

The genotype and allele frequencies of the *SPRN* gene were compared between the Dekalb White and Ross breeds by the chi-square (χ^2^) test and Fisher's exact tests using SAS 9.4 software (SAS Institute Inc., Cary, NC, USA). Linkage disequilibrium (LD) and haplotype analyses were performed using Haploview version 4.2 (Broad Institute, Cambridge, MA, USA).

### Multiple Sequence Alignments

The amino acid sequences of Sho were obtained from GenBank at the National Center for Biotechnology Information (NCBI), including those of humans (*Homo sapiens*, NP_001012526.2), cattle (*Bos taurus*, AAY83885.1), goats (*Capra hircus*, AGU17009.1), sheep (*Ovis aries*, NP_001156033.1), horses (*Equus caballus*, XP_023492126.1) and chickens (*Gallus gallus*, CAJ43796.1). The amino acid sequences of Sho were aligned using ClustalW with progressive alignment methods.

### Prediction of the N-Terminal Signal Peptide of Sho

The N-terminal signal peptide and cleavage site of Sho were predicted by SignalP 5.0 (https://services.healthtech.dtu.dk/service.php?SignalP-5.0). The prediction of SignalP 5.0 was performed according to a deep neural network-based method with conditional random field classification and optimized transfer learning.

### Prediction of Omega-Site and Signal Sequence of GPI-Anchor of the Sho

The omega-site and signal sequence of the GPI-anchor were predicted by PredGPI (http://gpcr.biocomp.unibo.it/predgpi/index.htm). The possibility of the signal sequence was evaluated based on a support vector machine (SVM). The omega-site was determined according to a hidden Markov model (HMM).

## Results

### Identification of Novel Polymorphisms of the *SPRN* Gene in Chickens

**Figure 1:**
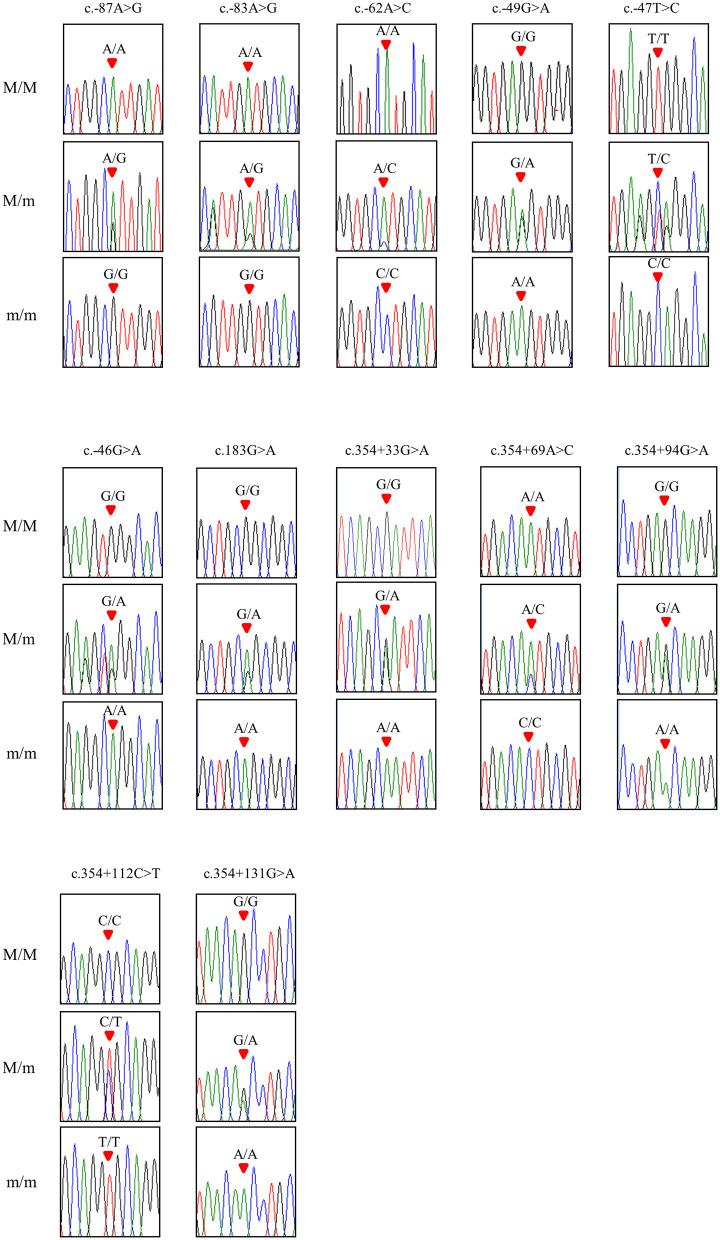
Electropherograms of 12 novel single-nucleotide polymorphisms (SNPs) of the shadow of prion protein gene (*SPRN*) found in chickens. The colors of the peaks indicate each base of the DNA sequence (green: adenine; red: thymine; blue: cytosine; black: guanine). The red arrows indicate the location of SNPs found in the present study. M/M, M/m and m/m indicate major homozygotes, heterozygotes and minor homozygotes, respectively.

To investigate chicken *SPRN* gene polymorphisms, we carried out PCR to amplify the open reading frame (ORF) region of the chicken *SPRN* gene. We identified a total of 12 novel SNPs, including c.183G>A (Ala61Ala) in the ORF region; 6 SNPs, including c.-87A>G, c.-83A>G, c.-62A>C, c.-49G>A, c.-47T>C and c.-46G>A in the upstream of the *SPRN* gene; and 5 SNPs, including c.354+33G>A, c.354+69A>C, c.354+94G>A, c.354+112C>T and c.354+131G>A in the downstream of the *SPRN* gene (Figure 1 and Table 1). Notably, c.-49G>A, c.-47T>C and c.354+33G>A were found in only Ross chickens, not Dekalb White chickens. The genotype and allele frequencies of polymorphisms of the chicken *SPRN* gene are described in Table 1. Among 12 SNPs, 9 SNPs, including c.-83A>G, c.-47T>C, c.-46G>A, c.183G>A (Ala61Ala), c.354+33G>A, c.354+69A>C, c.354+94G>A, c.354+112C>T and c.354+131G>A, showed significantly different genotype and allele frequencies of the *SPRN* gene between Dekalb White and Ross chickens.

**Table 1:** Genotype and allele frequencies of shadow of prion protein gene (*SPRN*) polymorphisms in chickens.

**Polymorphisms**	**Breeds**	**Total, n**	**Genotype frequencies, n**			***P*-value**	**Allele frequencies, n**		***P*-value**
			**MM**	**Mm**	**mm**		**M**	**m**	
c.-87A>G	Dekalb White	108	68	37	3	-	173	43	-
	Ross	110	71	35	4	0.8737	177	43	1.0
c.-83A>G	Dekalb White	108	81	24	3	-	186	30	-
	Ross	110	107	1	2	**<0.0001**	215	5	**<0.0001**
c.-62A>C	Dekalb White	108	101	4	3	-	206	10	-
	Ross	110	107	1	2	0.3042	215	5	0.1770
c.-49G>A	Dekalb White	108	74	31	3	-	179	37	-
	Ross	110	81	25	4	0.6403	187	33	0.5448
c.-47T>C	Dekalb White	108	108	0	0	-	216	0	-
	Ross	110	102	7	1	**0.0103**	211	9	**0.0036**
c.-46G>A	Dekalb White	108	108	0	0	-	216	0	-
	Ross	110	102	7	1	**0.0103**	211	9	**0.0036**
c.183G>A (Ala61Ala)	Dekalb White	108	58	49	1	-	165	51	-
	Ross	110	99	11	0	**<0.0001**	209	11	**<0.0001**
c.354+33G>A	Dekalb White	108	108	0	0	-	216	0	-
	Ross	110	99	11	0	**0.0008**	209	11	**0.0009**
c.354+69A>C	Dekalb White	108	78	4	26	-	160	56	-
	Ross	110	98	11	1	**<0.0001**	207	13	**<0.0001**
c.354+94G>A	Dekalb White	108	75	31	2	-	181	35	-
	Ross	110	99	9	2	**0.0001**	207	13	**0.0006**
c.354+112C>T	Dekalb White	108	41	37	30	-	119	97	-
	Ross	110	49	47	14	**0.0199**	145	75	**0.0209**
c.354+131G>A	Dekalb White	108	50	32	26	-	132	84	-
	Ross	110	101	7	2	**<0.0001**	209	11	**<0.0001**

*Bold texts indicate P < 0.05*.

We also investigated the linkage disequilibrium (LD) values among the 12 chicken *SPRN* polymorphisms with r^2^ values (Table 2). Notably, Dekalb white chickens showed a different distribution of strong LD than Ross chickens. Detailed information on the LD value is described in Table 2.

**Table 2:** Linkage disequilibrium (LD) among genetic polymorphisms of the *SPRN* gene in chickens.

**r^**2**^**	**c.-87A>G**	**c.-83A>G**	**c.-62A>C**	**c.-49G>A**	**c.-47T>C**	**c.-46G>A**	**c.183G>A**	**c.354+33** **G>A**	**c.354+69** **A>C**	**c.354+94** **G>A**	**c.354+112** **C>T**	**c.354+131 G>A**
c.-87A>G	-	**0.649**	0.195	**0.775**	-	-	0.028	-	0.199	**0.721**	0.175	0.157
c.-83A>G	0.096	-	0.301	**0.78**	-	-	0.203	-	**0.382**	**0.767**	0.112	0.201
c.−62A>C	0.096	**1.0**	-	0.235	-	-	0.004	–	0.104	0.191	0.024	0.053
c.-49G>A	**0.67**	0.132	0.132	-	-	-	0.09	-	0.266	**0.689**	0.123	0.214
c.-47T>C	0.01	0.001	0.001	0.008	-	-	-	-	-	-	-	-
c.-46G>A	0.01	0.001	0.001	0.008	**1.0**	-	-	-	-	-	-	-
c.183G>A	0.217	0.001	0.001	0.298	0.002	0.002	-	-	0.095	0.118	0.178	**0.384**
c.354+33 G>A	0.217	0.001	0.001	0.298	0.002	0.002	**1.0**	-	-	-	-	-
c.354+69 A>C	0.259	0.228	0.228	0.23	0.003	0.003	0.003	0.003	-	0.293	0.24	**0.487**
c.354+94 G>A	0.259	0.23	0.23	0.29	0.003	0.003	0.003	0.003	**0.447**	-	0.158	0.196
c.354+112 C>T	**0.43**	0.022	0.022	0.263	0.082	0.082	0.102	0.102	0.121	0.121	-	**0.403**
c.354+131 G>A	0.217	0.275	0.275	0.234	0.002	0.002	0.003	0.003	**0.542**	**0.418**	0.102	

*Bold texts indicate strong LD (r^2^ > 0.333). The above diagonal indicates the LD value in Dekalb White (n = 108). Below diagonal indicates the LD value in Ross chickens (n = 110)*.

We performed haplotype analysis of 12 genetic polymorphisms of the chicken *SPRN* gene (Table 3). Notably, Dekalb white showed different major haplotypes compared to Ross. In Dekalb White chickens, the AAAGTGGGAGTG haplotype was most frequently observed (40.6%), followed by the AAAGTGGGAGCG (13.0%), AAAGTGAGCGCA (12.0%) and GGAATGGGCACA (8.8%) haplotypes in the chicken *SPRN* gene (Table 3). In Ross chickens, the AAAGTGGGAGTG haplotype was most frequently observed (64.1%), followed by the AAAGTGGGAGCG (11.8%), GAAATGAAAGCG (5.0%) and AAAGCAGGAGCG (4.1%) haplotypes in the chicken *SPRN* gene (Table 3).

**Table 3:** Haplotype frequencies of 12 *SPRN* polymorphisms in Dekalb White and Ross chickens.

**Haplotype**	**Dekakb white (*n* = 216)**	**Ross (*n* = 220)**
AAAGTGGGAGTG	88 (0.406)	141 (0.641)
AAAGTGGGAGCG	28 (0.13)	26 (0.118)
AAAGTGAGCGCA	25 (0.12)	0
GGAATGGGCACA	19 (0.088)	0
AAAGTGAGAGCA	18 (0.085)	0
GAAATGAAAGCG	0	11 (0.05)
AAAGCAGGAGCG	0	9 (0.041)
GGCATGGGCACA	9 (0.042)	4 (0.018)
GAAATGGGAGCG	0	5 (0.023)
GAAGTGGGAGCG	5 (0.019)	8 (0.037)
AAAGTGAGAGTA	4 (0.017)	0
GAAATGGGCACA	0	3 (0.014)
AAAGTGAGAGCG	3 (0.014)	0
GAAATGGGAGCA	3 (0.014)	0
Others	14 (0.065)	13 (0.058)

### Multiple Sequence Alignments of Sho Among Species

**Figure 2:**
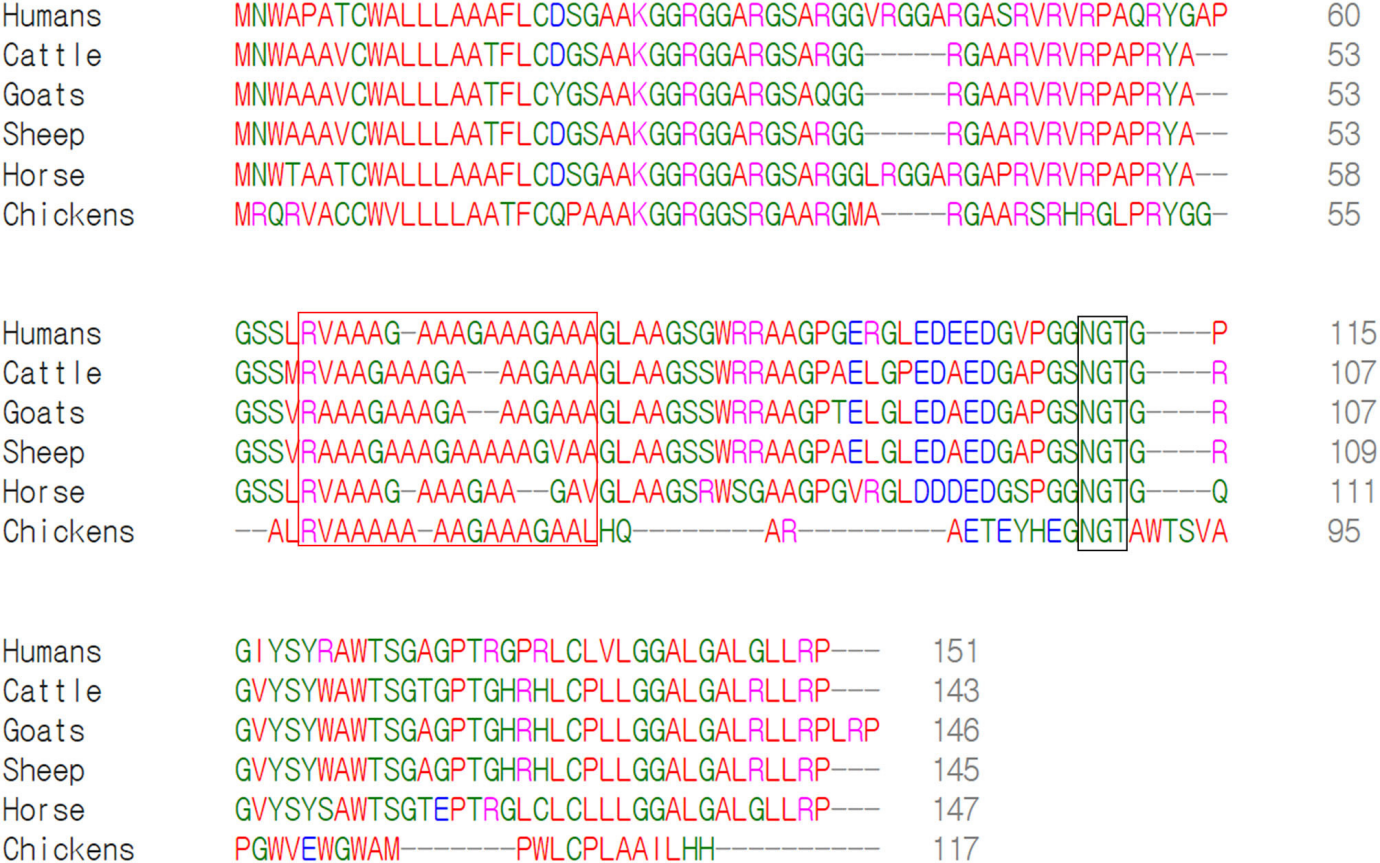
Multiple sequence alignments of amino acid sequences of the shadow of prion protein (Sho) in humans, cattle, goats, sheep, horses and chickens. Colors indicate the chemical properties of amino acids; blue: acidic; red: small and hydrophobic; magenta: basic; green: hydroxyl, sulfhydryl, amine and glycine. The red box indicates the interaction region of Sho with prion protein (PrP). The black box indicates the NXT glycosylation motif of Sho.

We performed multiple sequence alignments of amino acid sequences of Sho among humans, cattle, goats, sheep, horses and chickens (Figure 2). Among the 6 species, chicken Sho was the shortest (humans: 151, cattle: 143, goats: 146, sheep: 145, horses: 147; chickens: 117). Although the interaction regions between Sho and PrP (red box) and the NXT glycosylation motif (black box) were conserved among all species, sequence homology was extremely low in the N-terminal and C-terminal regions between mammals and chickens.

### Identification of Differences in the N-Terminal Signal Peptide of Sho Among Species

**Figure 3:**
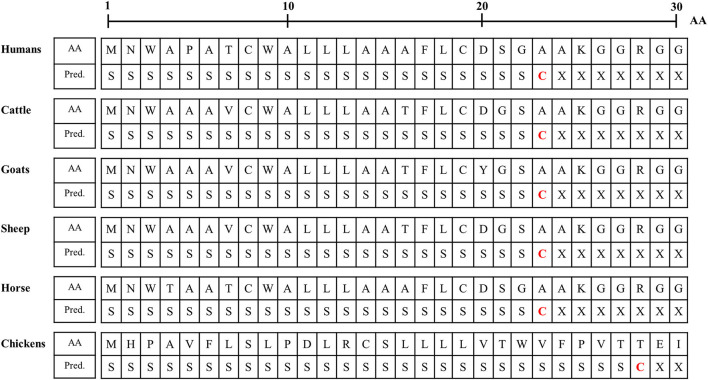
Signal peptide analysis of amino acid sequences of Sho in humans, cattle, goats, sheep, horses and chickens. AA, amino acids; Pred., Prediction; S, signal peptide; C, cutting site; X, nonsignal peptide.

We analyzed the N-terminal signal peptide of Sho among humans, cattle, goats, sheep horses and chickens. Detailed information on the amino acid sequences of the signal peptide is described in Figure 3. In brief, the length of the signal peptide of chicken (27 aa) was longer than that of humans, cattle, goats, sheep and horses (23 aa). In addition, the amino acid of the cutting site was threonine in chickens. However, the amino acid of the cutting site was alanine in mammals (Figure 3).

### Investigation of the Omega Site and Signal Sequence of the GPl-Anchor of Sho

We analyzed the omega-site and signal sequences of GPl anchor among humans, cattle, goats, sheep, horses, and chickens (Table 4). Among species, cattle showed the longest signal sequences of GPI anchors of the Sho (32 aa). Except for cattle (tyrosine), the amino acid of the omega-site of humans, goats, sheep, and horses was serine. Notably, omega sites were not predicted in chicken Sho.

**Table 4:** Prediction of the omega-site and signal sequences of the glycosylphosphatidylinositol (GPI)-anchor of Sho by PredGPI.

**Species**	**Omega-site**		**Signal sequence**		
	**Position**	**Amino acid**	**Position**	**Length**	**Protein sequence**
Humans	125	S	125–151	27	SGAGPTRGPRLCLVLGGALGALGLLRP
Cattle	112	Y	112–143	32	YWAWTSGTGPTGHRHLCPLLGGALGALRLLRP
Goats	117	S	117–146	30	SGAGPTGHRHLCPLLGGALGALRLLRPLRP
Sheep	119	S	119–145	27	SGAGPTGHRHLCPLLGGALGALRLLRP
Horses	117	S	117-147	31	SAWTSGTEPTRGLCLCLLLGGALGALGLLRP
Chickens	Not GPI-Anchored		-	-	-

### The Distributions of Genetic Polymorphisms of the *SPRN* Gene in Prion Disease-Resistant and Prion Disease-Susceptible Animals

**Figure 4:**
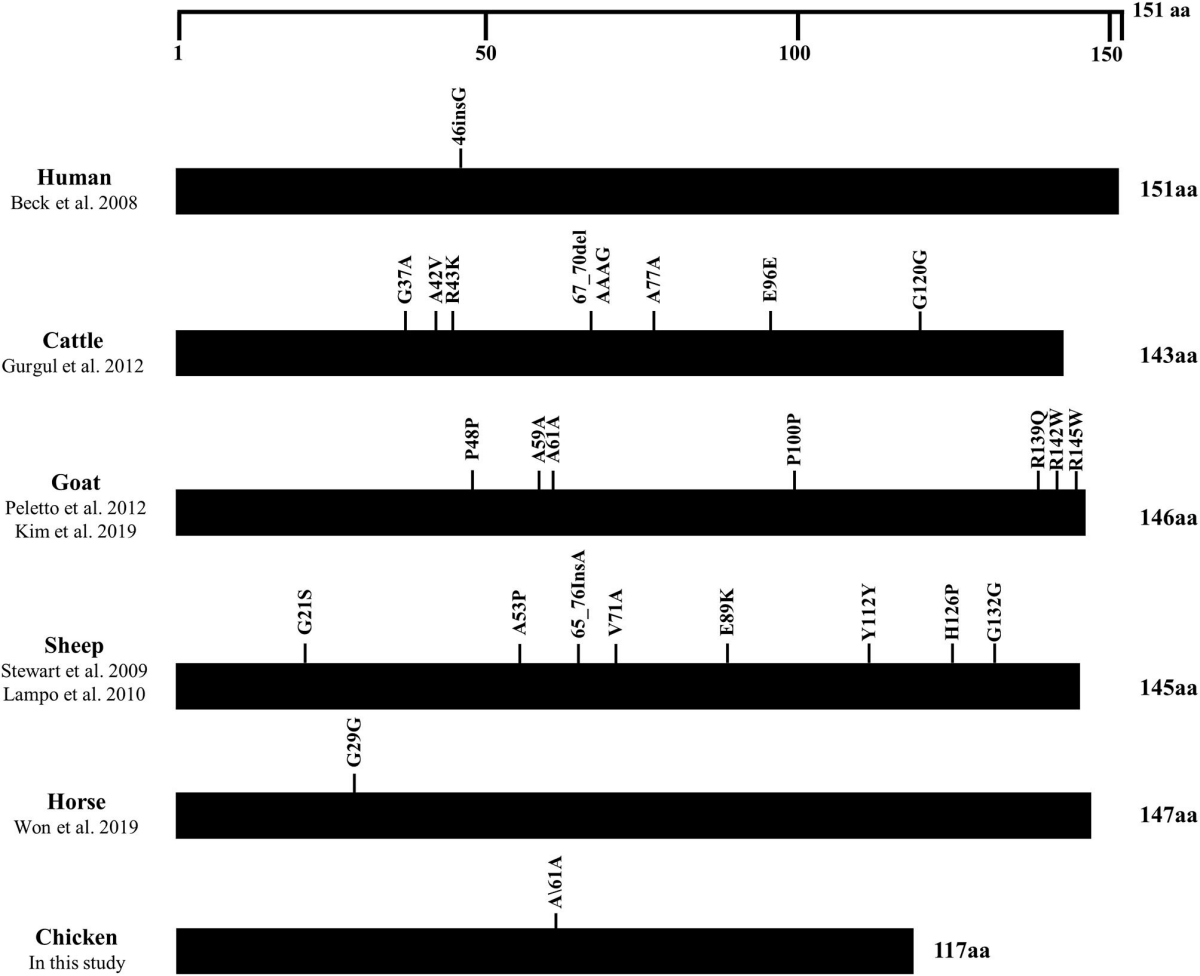
Distribution of genetic polymorphisms in the open reading frame (ORF) of the *SPRN* gene in various species. The figure shows the previously reported genetic polymorphisms of the *SPRN* gene in humans, cattle, goats, sheep, horses and chickens. The edged horizontal bar indicates the length of the amino acids in the *SPRN* gene.

We collected the polymorphisms found in the ORF of the *SPRN* gene in prion disease-resistant (horse, chickens) and prion disease-susceptible animals (human, cattle, goat and sheep) to find a difference in the distribution of genetic polymorphisms between these two groups. Notably, prion disease-susceptible animals had several genetic polymorphisms that cause amino acid changes in the ORF of the *SPRN* gene. However, only one synonymous SNP was found in prion disease-resistant animals, including horses and chickens (Figure 4).

## Discussion

In the present study, we found only one synonymous SNP, c.183G>A (Ala61Ala), in the ORF of the chicken *SPRN* gene in 2 chicken breeds, Dekalb White and Ross (Table 1). Although significant differences in the distributions of the genotype and allele frequencies of this SNP have been observed between these two breeds, the impact of SNP on chicken Sho is expected to be non-significant because the SNP is synonymous, which does not affect the structure of chicken Sho. Notably, previous studies have reported that only 3 synonymous SNPs that do not affect the structure of equine Sho were found in horses, a prion disease-resistant animal (13, 14). The absence of genetic polymorphisms in the ORF of the *SPRN* gene, which affects protein structure and expression level, seems to be a unique characteristic of prion disease-resistant animals, including horses and chickens. Further studies are needed to investigate whether these characteristics of prion-resistant animals are also observed in dogs, another prion-resistant animal. Except for the synonymous SNPs in the ORF of the chicken *SPRN* gene, we also found 6 SNPs in the adjacent region of the *SPRN* gene (Table 1). However, the exon structure of the chicken *SPRN* gene has not been confirmed thus far. Thus, further unknown region analysis of the exon structure of the chicken *SPRN* gene using 5' and 3' rapid amplification of cDNA ends (RACE) is highly desirable to investigate the impact of the SNPs on the chicken *SPRN* gene in the future.

Although the interspecies conserved PrP interaction domain and glycosylation motif were conserved in chicken Sho, significant heterogeneity was identified in the N- and C-terminal regions of chicken Sho compared to prion disease-related animals (Figure 2). Since the N- and C-terminal regions are related to the signal peptide of trafficking and GPI-anchor, respectively (19, 20), we analyzed the signal peptide of trafficking and GPI-anchor (Figure 3 and Table 4). Notably, the N-terminal signal peptide of chicken Sho was the longest among several species investigated, and the amino acid of the cutting site was threonine, unlike the interspecies-conserved amino acid of the cutting site, alanine (Figure 3). In addition, the signal peptide of the GPI-anchor was not predicted in chicken Sho (Table 4). These results indicate that chicken Sho may show different localization compared to prion-related animals. Previous studies have reported that PrP is located on lipid rafts and that experimental mislocalization and anchorless PrP disturbed the conversion process of PrP^Sc^ (21, 22). Since the conversion process of PrP^Sc^ occurs on lipid rafts, different localizations of chicken Sho may affect the conversion process of PrP^Sc^. Further investigation of the difference in localization of chicken Sho compared to other species is needed in the future.

## Conclusion

In summary, we found 7 novel SNPs, including 1 synonymous SNP in the ORF of the chicken *SPRN* gene. We found significantly different genotype, allele, and haplotype frequencies between Dekalb White and Ross chickens. We found that the interaction regions between Sho and PrP and the NXT glycosylation motif were conserved among all species; however, sequence similarity was extremely low in the N- and C-terminal regions between mammals and chickens. We found that chicken Sho has the longest N-terminal signal peptide, and the amino acids of the cutting site of chicken Sho are different from those of mammals. In addition, omega-site and signal sequences of the GPI-anchor were not predicted in only chickens. To the best of our knowledge, this is the first report of genetic polymorphisms of the chicken *SPRN* gene.

## Data Availability Statement

The original contributions presented in the study are included in the article/supplementary material, further inquiries can be directed to the corresponding author.

## Ethics Statement

The animal study was reviewed and approved by Institutional Animal Care and Use Committee (IACUC) of Jeonbuk National University.

## Author Contributions

Y-CK and B-HJ conceived and designed the experiment, analyzed the data, and wrote the paper. H-HK and Y-CK performed the experiments. All authors read and approved the final manuscript.

## Funding

This work was supported by the National Research Foundation of Korea (NRF) grant funded by the Korea government (MSIT) (2021R1A2C1013213, 2022R1C1C2004792). This research was supported by the Basic Science Research Program through the National Research Foundation (NRF) of Korea funded by the Ministry of Education (2017R1A6A1A03015876, 2021R1A6A3A010864).

## Conflict of Interest

The authors declare that the research was conducted in the absence of any commercial or financial relationships that could be construed as a potential conflict of interest.

## Publisher's Note

All claims expressed in this article are solely those of the authors and do not necessarily represent those of their affiliated organizations, or those of the publisher, the editors and the reviewers. Any product that may be evaluated in this article, or claim that may be made by its manufacturer, is not guaranteed or endorsed by the publisher.
